# Making neural networks more neural

**DOI:** 10.1016/j.patter.2026.101494

**Published:** 2026-02-13

**Authors:** Alan W. Freeman

**Affiliations:** 1 Save Sight Institute, University of Sydney, Sydney, NSW 2000, Australia

## Abstract

Deep neural networks (DNNs) are practical and effective but, despite the name, they lack biological validity. The recent study by Kang et al.^1^ in *Patterns* takes a step toward rectifying this deficit by hard-wiring receptive fields into the first layer of a visual DNN, and the authors show that their network can generalize across image types. Training on photographs, for example, resulted in good performance on sketches; conventional DNNs did not match this behavior.

## Teaser

Deep neural networks (DNNs) are practical and effective but, despite the name, they lack biological validity. The recent study by Kang et al. in *Patterns* takes a step toward rectifying this deficit by hard-wiring receptive fields into the first layer of a visual DNN, and the authors show that their network can generalize across image types. Training on photographs, for example, resulted in good performance on sketches; conventional DNNs did not match this behavior.

## Main text

Deep neural networks (DNNs) used for visual object recognition have a superficial resemblance to the visual pathway of the mammalian brain.^2^ Looking below the surface, however, reveals a distinct lack of features that are essential to the biological pathway. For example, neurons in the subcortical visual system are separated into parallel off-center and on-center pathways that respond to light with, respectively, decreased and increased activity. This dichotomy, ubiquitous across the evolutionary spectrum from flies^3^ to primates,^4^ is clearly a critical component of successful visual systems. Segregation into off- and on-pathways is typically absent in DNNs, representing a wasted opportunity. The most flexible and powerful object recognition machine resides in the primate brain: it is worth copying.

Kang et al.^1^ recently took a step toward making a visual DNN more lifelike. Using an AlexNet network,^5^ they replaced the first layer of their DNN with an array of Gabor filters. These filters, representing receptive fields in primary visual cortex, varied in preferred orientation, preferred spatial frequency, and spatial envelope. Filter characteristics remained fixed while the network was trained. Training and testing used the PACS dataset,^6^ which comprises four graphic domains (photograph, art, cartoon, and sketch), seven image classes (such as dog and elephant), and thousands of images for each domain and class.

The big advantage of the network with a Gabor front end was that it could generalize across domains. For example, training the network on photographic images resulted in strong object recognition on sketches without further training. Conventional networks, by contrast, fared poorly when switched between domains without extra training. The advantage of the Gabor-equipped network stemmed both from choosing a Gabor front end (other filters did not do as well) and from fixing the filters during training (trained Gabors yielded suboptimal object recognition).

Why does a Gabor DNN have greater applicability than a conventional DNN? Kang et al. analyzed this advantage by applying a principal components analysis to the activations of the first convolutional layer. They found that the mean number of dimensions required to account for the bulk of the variance was significantly less in the Gabor DNN than in a conventional DNN (about 6 versus 9). The authors argue that low-dimensional representations reduce the risk of overfitting to the training images and therefore increase the range of image types that the model can recognize.

Kang et al. found another important difference between conventional and Gabor DNNs by examining their ability to encode categorical information. The authors measured responses in the first fully connected layer. They again performed a dimensional analysis, this time using t-distributed stochastic neighbor embedding (t-SNE).^7^ Conventional DNNs tended to separate domains into separate clusters (for example, photographs versus sketches) without regard to class (such as dog versus elephant). The Gabor DNN produced a more useful result by separating classes into discrete clusters, as expected of efficient object recognition. The authors do not explain why the two types of model differ in this respect. Given that the first fully connected layer is downstream of the first convolutional layer, however, the superior categorization in the Gabor DNN could be inherited from the reduced overfitting at the front end of the model.

Finally, Kang et al. explored one more avenue in which their DNN could differ from conventional DNNs. They tested whether the Gabor DNN generalizes between images by encoding shape at the expense of texture. They passed their images through Frangi filters^8^ to emphasize edges and therefore shapes. To test for texture, they cut images into pieces and randomly shuffled the pieces. Presenting these images to DNNs trained on the original images showed that both types of DNN lost accuracy but differed in accuracy loss: the Gabor DNN lost less than the conventional DNN for shape-enhanced images but lost more for texture-enhanced images. It should be noted, however, that the study used a weak test for texture. A more realistic test would use textures such as bark, fur, and cloth. Such a test, however, is beyond the scope of the Kang et al. study.

Kang et al. used a bank of Gabor filters in the first layer of their DNN; filters differed in orientation, spatial frequency, and Gaussian envelope. There are at least two reasons why this bank is a good choice for object recognition.

1. An arbitrary image can be decomposed into a set of Gabor functions and reconstituted from these functions with arbitrary precision.^9^ To this extent, the use of Gabors can retain the essential information in an image.
2. The first convolutional layer in a conventional DNN is typically initialized with random weights and then trained with images. As the authors point out, this training can bias the weights to favor the training images. The use of Gabors avoids this bias.

Now that it has been shown that the inclusion of a biologically inspired layer improves the performance of a DNN, it is possible that the inclusion of other lifelike properties will improve performance even more. Kang et al. did not provide a mechanism giving rise to Gabor-shaped receptive fields even though such a mechanism exists. Soodak^10^ and Ringach^11^ showed that when off- and on-center inputs converge onto a cortical neuron, their antagonism results in a receptive field with two adjacent subfields, one for each input sign. This receptive field is well fitted by a Gabor function.^12^ It would be of great interest, therefore, to see whether a DNN with off- and on-pathways has a pattern recognition performance even better than that described by Kang et al.

## Declaration of interests

The author declares no competing interests.
